# Redox on the Clock: Sex-Dependent Dynamics of Xanthine Oxidoreductase Isoforms and Melatonin

**DOI:** 10.3390/ijms262311272

**Published:** 2025-11-21

**Authors:** Elżbieta Cecerska-Heryć, Martyn Zoń, Marta Budkowska, Natalia Serwin, Anna Michalczyk, Małgorzata Goszka, Aleksandra Polikowska, Bartosz Wojciuk, Barbara Dołęgowska

**Affiliations:** 1 Department of Laboratory Medicine, Pomeranian Medical University, 70-111 Szczecin, Poland; 2 Department of Analytical Medicine, Pomeranian Medical University, 70-111 Szczecin, Poland; 3 Department of Psychiatry, Pomeranian Medical University, 71-460 Szczecin, Poland; 4 Department of Immunological Diagnostics, Pomeranian Medical University of Szczecin, 70-111 Szczecin, Poland

**Keywords:** XOR, XDH, XD, oxidative stress assessment, chronobiology, chronotherapy

## Abstract

Melatonin, a key regulator of the circadian rhythm, exerts strong antioxidant effects by scavenging reactive oxygen species (ROS) and modulating enzymatic redox balance. Xanthine oxidoreductase (XOR), a molybdenum- and iron–sulfur-containing enzyme, catalyzes the oxidation of hypoxanthine to xanthine and xanthine to uric acid—the final steps of purine catabolism—serving as an important enzymatic source of ROS under physiological conditions. XOR exists in three interconvertible isoforms: xanthine dehydrogenase (XDH), which uses NAD^+^ as an electron acceptor; xanthine oxidase (XO), which transfers electrons to oxygen, producing superoxide and hydrogen peroxide; and an intermediate form (XDO) that reflects the redox-dependent interconversion between the two. This study aimed to evaluate temporal and sex-dependent variations in XOR isoforms and their relationship with melatonin levels in healthy individuals. Sixty-six volunteers (33 women aged 24–38 and 33 men aged 24–44) were examined. Blood samples were collected at 02:00, 08:00, 14:00, and 20:00. Serum melatonin was measured using ELISA, and XOR isoform activities were determined spectrophotometrically. Melatonin exhibited a precise 24 h rhythm with a nocturnal peak at 02:00 (~98 pg/mL) and a daytime nadir at 14:00 (~9 pg/mL). XO activity varied significantly (*p* < 0.01), showing an inverse correlation with melatonin in men (ρ = −0.52, *p* = 0.006), while XDO activity correlated positively with melatonin in women at 14:00 (ρ = 0.48, *p* = 0.01). These findings indicate sex-specific and time-dependent regulation of XOR isoforms, suggesting that redox homeostasis is modulated differently in men and women throughout the day. Understanding these dynamics may refine the interpretation of oxidative stress biomarkers and help optimize diagnostic and chronotherapeutic approaches in redox-related disorders.

## 1. Introduction

Living organisms display intrinsic 24 h rhythms that synchronize physiological and metabolic processes with the Earth’s rotation. These circadian rhythms regulate hormone secretion, sleep–wake cycles, metabolism, and cellular homeostasis [1].

Melatonin, primarily produced by the pineal gland, is a key hormonal regulator of biological rhythms. In healthy individuals, melatonin levels rise during the night and decline during the day, contributing to sleep–wake regulation. Beyond this role, melatonin exhibits potent antioxidant and anti-inflammatory properties. It directly scavenges reactive oxygen and nitrogen species, enhances the activity of major antioxidant enzymes (superoxide dismutase, glutathione peroxidase, catalase), inhibits lipid peroxidation, stabilizes mitochondrial function, and improves cellular resistance to oxidative stress [2,3]. Meta-analytic data confirm that melatonin supplementation increases antioxidant enzyme activity (SOD, GPx, TAC) and decreases lipid peroxidation markers such as malondialdehyde (MDA), supporting its role in redox regulation and cellular protection [4]. Oxidative stress occurs when the production of reactive oxygen species (ROS) exceeds the antioxidant capacity of cells, leading to damage to lipids, proteins, and nucleic acids. This imbalance contributes to the pathogenesis of cardiovascular, neurodegenerative, metabolic, and neoplastic diseases [1].

Xanthine oxidoreductase (XOR), encoded by the XDH gene on chromosome 2p23, plays a pivotal role in purine metabolism and redox regulation. It catalyzes the oxidation of hypoxanthine to xanthine, which is subsequently oxidized to uric acid. XOR exists in two primary interconvertible forms: xanthine dehydrogenase (XDH), which primarily uses NAD^+^ as an electron acceptor, and xanthine oxidase (XO), which reduces molecular oxygen to generate superoxide and hydrogen peroxide [5,6]. The conversion between these forms, mediated by redox-sensitive cysteine oxidation or limited proteolysis, can produce an intermediate isoform (XDO) [6]. While XO contributes to ROS generation, its substrates (hypoxanthine and xanthine) and the final product, uric acid, also possess antioxidant properties—scavenging singlet oxygen, hydroxyl radicals, and peroxynitrite. Thus, XOR exhibits a dual redox nature, functioning as both a prooxidant and an antioxidant enzyme depending on its isoform balance and redox environment [7,8]. Emerging evidence indicates that circadian regulation may influence blood modulation of xanthine oxidoreductase activity. Clinical studies have shown diurnal variations in serum uric acid and XOR activity, with higher values observed in the morning, suggesting clock-dependent metabolic regulation [9,10]. Experimental data also demonstrate circadian control of oxidative enzymes, including superoxide dismutase and glutathione peroxidase, which contain clock-responsive elements in their promoter regions [11].

Collectively, these findings point to a complex interaction among circadian rhythms, oxidative stress, and purine metabolism. Disruption of circadian stability—including the age-related decline in melatonin production—has been associated with impaired redox balance and increased disease susceptibility [12,13,14]. Melatonin, as a regulator of biological timing and a potent antioxidant, may indirectly modulate XOR-related pathways. However, the mechanisms underlying the regulatory relationship between melatonin and XOR remain unclear, and current evidence supports only correlational, not causal, relationships.

Therefore, the present study aimed to investigate the circadian modulation of xanthine oxidoreductase activity and its isoforms in healthy individuals, as well as their association with melatonin levels and oxidative balance. Specifically, this research sought to

1. Characterize circadian variations in xanthine oxidoreductase isoforms and their metabolic products.
2. Evaluate relationships between melatonin concentration, oxidative stress markers, and the balance of XOR isoforms.

We hypothesize that the activity of xanthine oxidoreductase follows a circadian rhythm that reflects coordinated regulation of purine metabolism and redox homeostasis, potentially influenced by daily hormonal and metabolic fluctuations.

## 2. Results

### 2.1. Analysis of Biochemical, Morphological, and Mineral Parameters

Table 1 displays the results of biochemical, morphological, and mineral parameters. The values are presented as arithmetic means along with standard deviations for both women and men within the reference range. Significant statistical differences between the sexes were observed for the following parameters: RBC, HGB, HCT, TAG, creatinine, and uric acid. In these cases, the significance level (*p*-value) was not significant (*p* > 0.05).

### 2.2. Analysis of Melatonin Concentration at Different Times of the Day

Melatonin assays revealed apparent diurnal variations in hormone levels throughout the day. The average melatonin concentration peaked at 02:00, reaching 99.5 ± 11.2 pg/mL in women and 97.1 ± 11.7 pg/mL in men, indicating a pronounced nocturnal surge. In contrast, the lowest concentrations were observed at 14:00, decreasing to 8.7 ± 2.5 pg/mL in women and 8.0 ± 2.6 pg/mL in men, confirming a marked day–night variation (Supplementary Tables S1 and S2).

Statistical analysis using the Friedman ANOVA test revealed significant differences in melatonin levels across sampling times (*p* < 0.001 for both sexes). Underscoring the robustness of the circadian pattern. No statistically significant differences between women and men were observed at any specific time point (all *p* > 0.05), indicating similar rhythms and amplitudes of melatonin secretion across the sexes. Kendall’s coefficient of concordance demonstrated high within-group consistency, with values of W = 0.936 (*p* < 0.001) for women and W = 0.950 (*p* < 0.001) for men.

**Figure 1:**
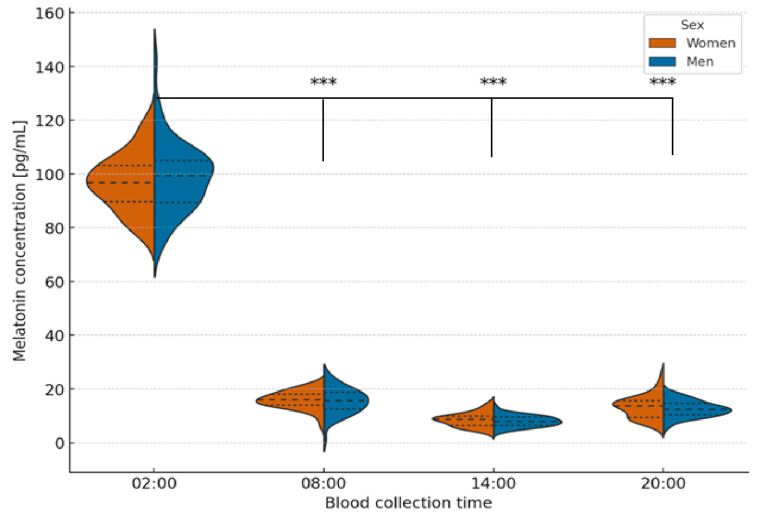
Violin plots showing the distribution, median, and interquartile range of serum melatonin concentrations in men and women according to blood collection time. Friedman ANOVA revealed statistically significant differences in melatonin concentrations across all time points in both sexes (*p* < 0.001). Melatonin levels peaked at 02:00 and were significantly higher compared with other time points (*p* < 0.001, Bonferroni-adjusted). Asterisks (*** *p* < 0.001) indicate significant differences relative to 02:00.

A repeated-measures ANOVA followed by a Bonferroni correction revealed significant differences across all time points (*p* < 0.0001 for pairwise comparisons). Further supporting the strong circadian regulation of melatonin secretion. MANOVA analysis also showed a significant overall time effect (*p* < 0.001). Still, no significant interaction between sex and time, confirming that the temporal pattern of melatonin secretion was consistent in both sexes, with peak concentrations around 02:00 and a sharp decline during daytime hours (Figure 1).

### 2.3. Analysis of XOR Isoform Concentrations at Different Times of Day

Based on dehydrogenase activity measurements, a statistical analysis was conducted. The median XDH activity in men was lowest at 8:00, recording a value of 5.51 mU/mL, and highest at 2:00, reaching 7.47 mU/mL. In women, XDH activity varied; the lowest was at 2:00 at 6.17 mU/mL, while the highest, similar to that in men, was at 14:00 at 7.39 mU/mL (Supplementary Tables S3 and S4).

However, the Friedman ANOVA test did not identify any statistically significant differences in XDH activity across the various blood collection times within the study population, with *p*-values of 0.14 for men and 0.16 for women. Kendall’s coefficient of concordance was recorded at 0.12 for men and 0.07 for women.

**Figure 2:**
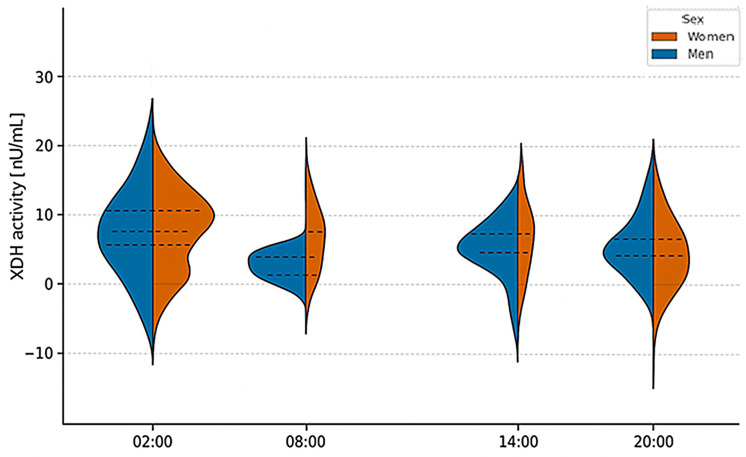
Violin plots showing the distribution, median, and interquartile range of xanthine dehydrogenase (XDH) activity in men and women according to blood collection time. Friedman ANOVA revealed no statistically significant differences in XDH activity across time points in either sex (*p* > 0.05). MANOVA confirmed no significant interaction between sex and time of collection (*p* = 0.12, ns). Values are presented as mean ± SD.

Additionally, a MANOVA test did not reveal statistically significant differences in dehydrogenase activity between the sexes (*p*-value = 0.12), exceeding the significance threshold of α = 0.05. Detailed results are presented in Supplementary Tables S3 and S4 and Figure 2.

Furthermore, we investigated whether there was a statistically significant correlation between XDH activity and melatonin concentration. Spearman’s rank correlation coefficient indicated no significant correlation in either women or men.

Based on the results of dehydrogenase–oxidase activity measurements, a detailed statistical analysis was conducted. The median XDO activity in men was lowest at 20:00 (1.09 mU/mL) and highest at 14:00 (19.7 mU/mL). In women, the values differed: the lowest XDO activity was observed at 14:00 (5.38 mU/mL), while the highest was recorded at 20:00 (13.4 mU/mL) (Supplementary Tables S5 and S6). The Friedman ANOVA test revealed statistically significant differences in XDO activity among men (*p* = 0.001), with Kendall’s coefficient of concordance at 0.49. The analysis was further extended with a Bonferroni correction for multiple comparisons, which confirmed statistically significant differences between 2:00 and 20:00 (*p* = 0.016), 2:00 and 8:00 (*p* = 0.023), 8:00 and 20:00 (*p* = 0.003), and 14:00 and 20:00 (*p* = 0.01).

**Figure 3:**
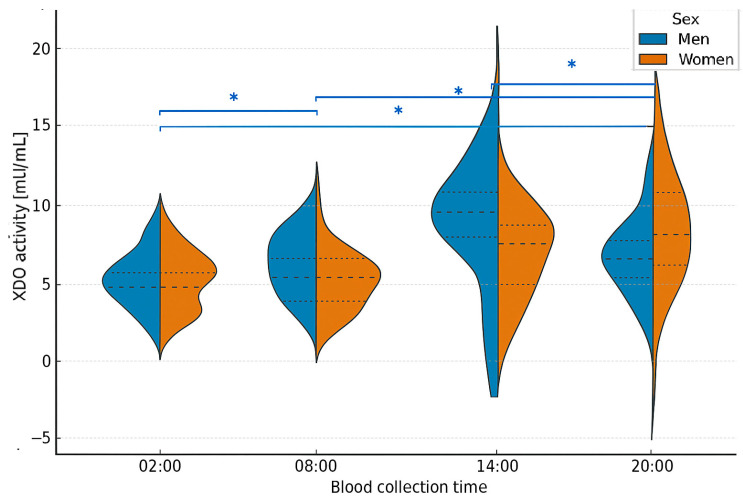
Violin plots showing the distribution, median, and interquartile range of xanthine dehydrogenase/oxidase (XDO) activity in men and women according to blood collection time. Friedman ANOVA revealed significant time-dependent differences in men (*p* < 0.001), but not in women (*p* = 0.054, ns). Post hoc Bonferroni correction confirmed significant differences in men between 02:00 and 20:00 (*p* = 0.016), 02:00 and 08:00 (*p* = 0.023), 08:00 and 20:00 (*p* = 0.003), and 14:00 and 20:00 (*p* = 0.01). MANOVA confirmed overall between-sex differences (*p* < 0.001). Asterisks * indicate statistically significant differences after Bonferroni correction.

In women, however, the Friedman ANOVA test did not show statistically significant differences, although the *p*-value approached significance (*p* = 0.054), with Kendall’s coefficient of concordance at 0.18. Additionally, a MANOVA test revealed statistically significant differences in XDO activity between sexes (*p* < 0.001), well below the α = 0.05 significance threshold (Figure 3). Finally, the correlation between XDO activity and melatonin concentration was examined by sex. Spearman’s rank correlation coefficient did not reveal statistical significance in men. In contrast, in women, a significant negative correlation was found between XDO activity and melatonin concentration at 14:00 (*p* = 0.01), indicating that lower melatonin levels were associated with reduced XDO activity. This suggests that the daytime decline in melatonin may parallel a decrease in XOR-dependent antioxidant capacity, reflecting the interplay between circadian and redox regulation.

Based on the results of oxidase activity measurements. a statistical analysis was conducted. In men, the median xanthine oxidase (XO) activity was lowest at 2:00, measured at 4.33 mU/mL (Supplementary Tables S7 and S8). The highest value was at 2:00, with a measurement of 25.5 mU/mL. In contrast, women showed different values, with the lowest XO activity recorded at 14:00 (6.14 mU/mL) and the highest at 2:00 (12.92 mU/mL).

The Friedman ANOVA test revealed statistically significant differences in XO activity across various sample collection times. The *p*-values from this test were 0.002 for men and 0.046 for women, indicating significant differences in both sexes. Kendall’s coefficient of concordance was 0.19 for men and 0.13 for women.

**Figure 4:**
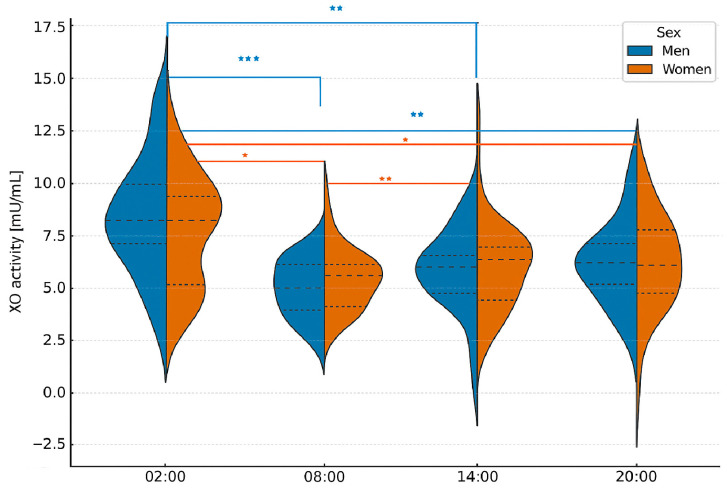
Violin plots showing the distribution, median, and interquartile range of xanthine oxidase (XO) activity in men and women according to blood collection time. Friedman ANOVA revealed significant time-dependent differences in both men (*p* = 0.002) and women (*p* = 0.046). Post hoc Bonferroni correction confirmed significant pairwise differences: Men: 02:00 vs. 08:00 (*p* = 0.0002), 02:00 vs. 14:00 (*p* = 0.001), 02:00 vs. 20:00 (*p* = 0.007); Women: 02:00 vs. 08:00 (*p* = 0.025), 02:00 vs. 14:00 (*p* = 0.0017), 02:00 vs. 20:00 (*p* = 0.017). MANOVA confirmed significant overall differences between sexes (*p* = 0.0002). Asterisks indicate the level of statistical significance after Bonferroni correction: * *p* < 0.05; ** *p* < 0.01; *** *p* < 0.001.

Using the Bonferroni correction for multiple comparisons, we confirmed significant differences in men between 2:00 and 8:00 (*p* = 0.0002). 2:00 and 14:00 (*p* = 0.001), and 02:00 and 20:00 (*p* = 0.007). In women, significant differences were observed between 02:00 and 08:00 (*p* = 0.025). 2:00 and 14:00 (*p* = 0.0017), and 2:00 and 8:00 (*p* = 0.017). Furthermore. MANOVA analysis indicated a statistically significant difference in XO activity between the sexes (*p*-value = 0.0002), which is below the established significance threshold of α = 0.05, suggesting apparent differences between the groups (Figure 4).

Finally, we examined the correlation between XO activity and melatonin concentration by sex. Spearman’s rank correlation coefficient indicated a statistically significant correlation in men between XO activity and melatonin levels measured at 2:00 (*p* = 0.006). At this time. XO activity was inversely proportional to melatonin concentration; melatonin levels were highest when XO activity was lowest. In women, a significant correlation was found between XO and melatonin at 14:00 (*p* = 0.01).

**Figure 5:**
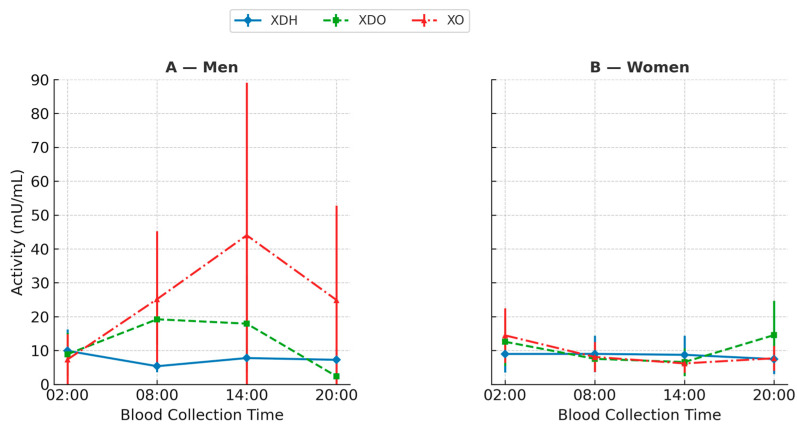
Mean activity of xanthine oxidoreductase (XOR) isoforms—xanthine dehydrogenase (XDH), xanthine dehydrogenase/oxidase (XDO), and xanthine oxidase (XO)—in men (**A**) and women (**B**) according to blood collection time. Values are presented as mean ± standard deviation, with offset error bars to minimize overlap between time points. Separate Y-axes were applied for men and women solely to improve visual clarity, as the absolute activity values differed between sexes but remained within comparable biological ranges. Statistical analysis using one-way ANOVA revealed significant differences between isoforms (*p* < 0.001). Post hoc Tukey’s test identified specific pairwise differences (*p* < 0.05), as indicated in the figure.

Finally, it was examined whether the activities of individual isoforms differed between the sexes. A one-way ANOVA revealed differences in the activities of the XOR isoforms. For both men and women, the *p*-value was <0.001, indicating a statistically significant difference between at least two of the three XOR isoform activities (Figure 5).

### 2.4. Circadian Profiles of XOR Isoforms and Melatonin-Cosinor Analysis

The combined 24 h cosinor analysis revealed strong, highly significant rhythmicity in plasma melatonin (global *p* < 10^−6^), with nearly identical acrophases in women and men (≈3.8–3.9 h).

In contrast, none of the xanthine oxidoreductase isoforms (XDH, XDO, XO) exhibited statistically significant 24 h rhythmicity (all *p* > 0.1). The estimated amplitudes were low (XDH = 2.21 mU/mL; XDO = 1.26 mU/mL; XO = 1.34 mU/mL) and associated with weak model fits (R^2^ < 0.03).

**Figure 6:**
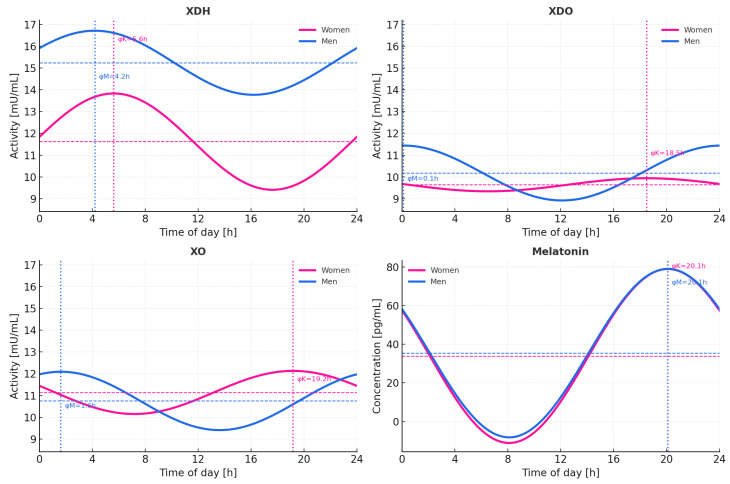
Circadian cosinor analysis of xanthine oxidoreductase isoforms (XDH, XDO, XO) and melatonin in women and men. Cosinor analysis (24 h) showed non-significant rhythms for XDH (*p* = 0.20; amp = 2.21 mU/mL). XDO (*p* = 0.39; amp = 1.26 mU/mL), and XO (*p* = 0.34; amp = 1.34 mU/mL). A significant circadian rhythm was observed for melatonin (*p* < 0.001; amp = 45.08 pg/mL). Estimated acrophases were: XDH—5.6 h (women)/4.2 h (men); XDO—18.5 h (women)/0.1 h (men); XO—20.2 h (women)/1.2 h (men); melatonin—20.1 h (women)/20.2 h (men). Dashed lines mark acrophases. XDH—xanthine dehydrogenase; XDO—dehydrogenase–oxidase form; XO—xanthine oxidase.

Acrophase estimates showed significant interindividual variability, with approximate peaks at 5.6 h (women) and 4.2 h (men) for XDH. 18.5 h (women) and 0.1 h (men) for XDO, and 20.2 h (women) and 1.2 h (men) for XO. Sex-phase differences (Δφ) were minimal for melatonin (−0.03 h) and variable but nonsignificant for XOR enzymes (Δφ ≈ −1 to −18 h). Detailed results are summarized in Table 2 and illustrated in Figure 6.

Cosinor analysis of the total study group did not reveal significant 24 h rhythmicity in any of the xanthine oxidoreductase (XOR) isoforms (Table 3). Xanthine dehydrogenase (XDH) exhibited only a weak oscillatory trend (MESOR = 13.43 mU/mL; amplitude = 1.85 mU/mL; *p* = 0.094), suggesting limited diurnal variation. Both the intermediate dehydrogenase–oxidase form (XDO) and xanthine oxidase (XO) demonstrated even lower amplitudes (0.62 and 0.78 mU/mL, respectively) and poor model fit (R^2^ ≤ 0.01), indicating a lack of consistent circadian pattern in their plasma activity. In contrast, melatonin displayed a robust, statistically significant 24 h rhythm (*p* < 1 × 10^−6^), characterized by a high amplitude (44.38 pg/mL) and a strong model fit (R^2^ = 0.66). The estimated acrophase (3.85 rad; approximately 22:00 h) corresponded to the expected nocturnal peak in melatonin secretion.

## 3. Discussion

The present study demonstrated distinct sex-related differences in several biochemical and hematological parameters while confirming normal values across all measured indices. Significant differences between women and men were observed for RBC. HGB. HCT. TAG, creatinine, and uric acid levels. The mean RBC, hemoglobin, and hematocrit values were higher in men, consistent with known physiological differences related to body mass, blood volume, and the stimulatory effect of testosterone on erythropoiesis [15,16,17,18,19].

Triglyceride (TAG) levels also exhibited sex-dependent variation, averaging 92 ± 19 mg/dL in women and 111 ± 35 mg/dL in men, both below the diagnostic threshold of 150 mg/dL [15]. These findings reflect the influence of metabolic and hormonal factors, as well as lifestyle factors such as diet and physical activity, which are known to modulate lipid metabolism. TAG levels also display seasonal variability, typically showing inverse correlations with HDL cholesterol, which is physiologically higher in premenopausal women [20].

Differences in creatinine and uric acid concentrations followed expected sex-related trends, reflecting variations in muscle mass, protein intake, and renal urate clearance. Mean creatinine values were 1.0 ± 0.2 mg/dL in women and 1.1 ± 0.2 mg/dL in men. At the same time, uric acid averaged 4.5 ± 0.7 mg/dL in women and 5.3 ± 1.0 mg/dL in men, remaining within reference ranges for both sexes [14,21,22,23].

Melatonin levels displayed a robust circadian pattern, peaking at 2:00, and reaching their lowest values around 14:00 in both sexes. Statistical analysis confirmed significant differences between sampling times (*p* < 0.001), consistent with previous research demonstrating nocturnal melatonin surges in healthy individuals [24,25,26]. No significant differences were observed between women and men at any time point, suggesting similar rhythmic secretion and amplitude. The maintenance of physiological sleep–wake cycles during the experiment was further supported by this consistent circadian pattern.

The circadian rhythm plays a crucial role in coordinating numerous physiological processes, including hormone secretion, cellular renewal, and metabolism [11]. Disturbances of this rhythm have been linked to metabolic, cardiovascular, and neurodegenerative diseases [12]. Increasing evidence indicates that oxidative stress and circadian regulation are interconnected, though the causal mechanisms remain under investigation [13].

In the present study, the activities of xanthine oxidoreductase (XOR) isoforms displayed clear time- and sex-dependent variations, indicating potential circadian regulation of purine metabolism and redox homeostasis. The activity of xanthine dehydrogenase (XDH) peaked at 2:00 in both sexes, consistent with an enhanced nocturnal antioxidant response. In contrast, xanthine oxidase (XO) activity showed the opposite pattern, with the highest values at 14:00 in men and 2:00 in women. Although the Friedman ANOVA did not confirm statistically significant fluctuations in XDH activity, the observed nocturnal increase suggests a physiological shift toward antioxidant predominance during the rest phase. In contrast, XDO and XO activities varied significantly across time points—particularly in men—indicating sex-specific regulation of the enzymatic interconversion between XDH and XO (*p* = 0.001 and *p* = 0.002, respectively).

These findings support the hypothesis that oxidative metabolism is rhythmically modulated, aligning with diurnal energy expenditure and hormonal activity. Sex hormones are likely to play an essential role in these patterns. Estrogens have been shown to downregulate XO activity and reduce ROS generation by upregulating antioxidant enzyme expression, including SOD and GPx [27]. Furthermore, estrogen-induced reactive oxygen species can trigger epigenetic reprogramming that modulates redox-sensitive gene expression, indicating a complex feedback between estrogen signaling and oxidative metabolism [28]. In contrast, testosterone may stimulate oxidative pathways and promote the conversion of XDH to XO via androgen receptor-mediated transcriptional signaling [29,30]. These mechanisms may explain the higher daytime XO activity observed in men and the delayed or inverted pattern in women, where estrogens maintain greater antioxidative potential. Furthermore, circadian clock genes such as BMAL1 and CLOCK directly regulate the transcription of antioxidant defense systems and hepatic metabolic enzymes, suggesting that temporal changes in XOR activity may also result from clock-controlled redox and energy metabolism gene expression [31,32]. These mechanisms may explain the higher daytime XO activity observed in men and the delayed or inverted pattern in women, where estrogens maintain greater antioxidative potential.

The observed inverse relationship between XO activity and melatonin concentration at 2:00 in men (*p* = 0.006) further supports the interplay between circadian and oxidative systems. Melatonin can modulate XOR activity through several mechanisms—direct inhibition of xanthine oxidase-mediated ROS formation, upregulation of XDH expression, and modulation of mitochondrial redox state [33,34,35,36]. Notably, the magnitude of these effects appears to differ between the sexes: studies indicate that melatonin’s antioxidative efficacy is enhanced by estrogens and reduced by androgens, suggesting that women may experience greater enzymatic suppression of prooxidant XOR forms during the night [37,38].

Comparable trends have been reported by Shimizu et al. [7], who demonstrated diurnal variations in serum uric acid, xanthine, and XOR activity in men with coronary artery disease. Morning peaks suggest enhanced oxidative metabolism upon waking. Our data in healthy individuals confirm this pattern and extend it by highlighting sex-dependent oscillations in XOR isoforms.

Population studies have also shown that XOR activity is generally higher in men, correlating with metabolic and cardiovascular risk [39,40]. Elevated XOR levels are associated with increased blood pressure independently of uric acid concentrations, suggesting that the enzyme contributes to vascular tone regulation and endothelial ROS signaling [41,42]. The higher daytime XO activity observed in men in our study may therefore reflect their greater metabolic load and oxidative susceptibility, consistent with epidemiological data [43].

Clinical evidence further supports the pathophysiological significance of XOR activity, linking its elevation to adverse outcomes in heart failure and progression of chronic kidney disease (CKD) [44,45,46]. Moreover, nocturnal hypoxia has been shown to increase XOR activity and oxidative stress markers, underscoring the potential contribution of circadian misalignment to cardiometabolic risk [7]. Recent chronobiological studies reveal that sex differences in circadian regulation manifest at multiple levels, including the intrinsic period of clock-controlled physiology and redox gene expression. For example, women show shorter intrinsic circadian periods and stronger antioxidant responses compared to men [47,48,49]. These sex-specific circadian and redox dynamics may help explain the differences we observed in XOR isoform activity between men and women.

Taken together, these findings indicate that XOR isoform activity shows both circadian and sex-specific variation, reflecting the complex interactions among hormonal regulation, oxidative metabolism, and biological timing. Such differences may serve as early indicators of disrupted redox homeostasis and increased cardiometabolic vulnerability. Integrating the assessment of XOR isoforms. XO activity and uric acid concentrations could provide a more comprehensive understanding of time-dependent redox regulation and its physiological and clinical relevance. Furthermore, recognition of sex-dependent rhythmic modulation underscores the need for chronobiology-informed approaches to studying oxidative metabolism and to developing personalized therapeutic strategies.

In a broader biological context. XOR is a key hydroxylase in purine metabolism, catalyzing the oxidation of hypoxanthine to xanthine and subsequently to uric acid (UA). It exists as a homodimeric enzyme capable of interconverting between distinct isoforms: XDH, which is predominant in physiological conditions, and XO, generated via oxidation of cysteine residues or limited proteolysis during tissue stress [50,51,52]. These isoforms exert opposing effects on redox balance: XDH functions as an antioxidant by utilizing NAD^+^, while XO produces ROS by reducing molecular oxygen [53,54]. XDH/XO (XDO) can use both NAD^+^ and O_2_ as electron acceptors, reflecting transitional enzyme states during redox adaptation [10,55].

Structural and mechanistic studies have further established XOR as more than a metabolic enzyme: it acts as a regulator of redox signaling and inflammation, contributing to both physiological defense and pathological oxidative stress [56,57,58]. Under conditions such as ischemia–reperfusion injury, the conversion from XDH to XO promotes ROS generation, exacerbating oxidative damage but also supporting antimicrobial defense responses [59].

The last isoform analyzed was XO, the prooxidant form. In men, median XO activity ranged from 4.33 mU/mL at 2:00 to 25.5 mU/mL at 14:00, while in women it ranged from 6.14 mU/mL at 14:00 to 12.92 mU/mL at 2:00. Friedman ANOVA confirmed significant differences in both sexes (*p* = 0.002 for men, *p* = 0.046 for women). Notably, XO activity was lowest at 2:00 in men but highest at 2:00 in women, opposite to XDH dynamics, consistent with their complementary roles in maintaining redox balance.

Comparable findings were reported in men with coronary artery disease, where plasma XOR, UA, and xanthine increased from evening to morning with a midday decline [7]. Nocturnal hypoxia and sleep-disordered breathing have also been linked to overnight rises in XOR activity [60]. Large cohorts confirm higher XOR activity in men, which is associated with greater cardiometabolic burden [61,62], elevated blood pressure independent of UA [50], and increased cardiovascular risk [63,64]. This underscores the clinical significance of the observed diurnal patterns.

To further explore whether the observed fluctuations in XOR activity followed a genuine circadian rhythm, a Cosinor analysis was performed. This model confirmed a strong 24 h rhythm of melatonin secretion (*p* < 10^−6^), validating the integrity of the experimental design and participants’ circadian stability. In contrast, none of the XOR isoforms (XDH, XDO, XO) showed statistically significant rhythmicity (*p* > 0.1), and their estimated amplitudes were low, suggesting that daily variations in enzymatic activity were modest and highly individualized. These findings are in line with previous observations showing that not all oxidative enzymes exhibit robust circadian oscillations in humans [65,66].

The near-significant trend observed for XDH (*p* = 0.094) might indicate a weak endogenous rhythm, consistent with its antioxidative role and earlier studies describing partial circadian control of redox enzymes such as superoxide dismutase, catalase, and glutathione peroxidase [9,65,67]. However, the overall low amplitude and poor model fit (R^2^ < 0.03) imply that XOR activity in healthy adults is primarily influenced by metabolic and hormonal factors rather than by intrinsic circadian mechanisms [68,69].

Interestingly, the timing of minimal XOR activity in men coincided with the nocturnal melatonin peak, further supported by the inverse relationship between XO activity and melatonin concentration at 2:00 (*p* = 0.006). This finding suggests that melatonin may exert a transient inhibitory effect on oxidative forms of XOR, consistent with reports of melatonin-mediated suppression of xanthine oxidase-dependent ROS production [34,35,36,37,38,39,40,41,42,43,44,45,46,47,48,49,50,51,52,53,54,55,56,57,58,59,60,61,62,63,64,65,66,67,68,69,70]. Melatonin has been shown to modulate oxidative metabolism through direct scavenging, regulation of mitochondrial redox state, and transcriptional control of antioxidant enzymes [24,25,65,71].

Taken together, the Cosinor analysis supports the conclusion that, although melatonin exhibits a clear and robust circadian rhythm, the activity of xanthine oxidoreductase isoforms remains relatively stable across the 24 h. Such stability likely reflects a physiological mechanism maintaining purine catabolism and redox homeostasis, independent of short-term circadian oscillations [72,73].

The subtle, sex-dependent differences observed here warrant further investigation, particularly in clinical settings where circadian misalignment and oxidative stress coexist [74]. 

## 4. Materials and Methods

### 4.1. Characteristics of Study Groups

The study group consisted of 66 healthy individuals with a balanced gender distribution: 33 women aged 24 to 38 years and 33 men aged 24 to 44 years. To maintain the integrity and homogeneity of the study population, each participant was required to meet specific eligibility criteria. A comprehensive questionnaire was administered to gather information on lifestyle factors, including physical activity, sleep duration, and medication or dietary supplement use.

For female participants, blood collection was scheduled during the follicular phase (days 5–10 of the menstrual cycle) to minimize hormonal variability. Women with irregular cycles or using hormonal contraception were excluded.

Inclusion Criteria:

- Healthy adult women aged 24 to 38 years and men aged 24 to 44 years.
- No chronic diseases and no long-term pharmacotherapy.
- No antibiotic treatment within the recent period.
- No use of painkillers in the two weeks before sample collection.
- No hormone replacement therapy.
- For women: not using oral contraceptives and not pregnant.
- Normal results for laboratory analyses, including complete blood count, glucose, albumin, total protein, creatinine, uric acid, lipid profile (total cholesterol, HDL, LDL, TAG), and mineral parameters (total calcium, inorganic phosphorus, magnesium).
- Regular daily rhythm (no night or shift work).
- Written informed consent for participation.

Exclusion Criteria:

- Smoking
- Night-shift or rotating-shift work that disrupts circadian rhythm.
- Ongoing pharmacotherapy for chronic diseases.
- Current or recent (within two weeks) antibiotic therapy.
- Use of analgesics in the two weeks preceding sample collection.
- Hormone replacement therapy or use of oral contraceptives.
- Pregnancy (for women participants).
- Abnormal laboratory findings indicating metabolic disturbances or chronic illness.
- Withdrawal of consent at any point during the study.

To verify participants’ health status, each individual underwent a complete peripheral blood count and biochemical assessment. This included testing for glucose, albumin, total protein, creatinine, uric acid, lipid profile (total cholesterol, HDL, LDL, TAG), and mineral levels (calcium, phosphorus, magnesium). To further support circadian rhythm regulation, participants were provided with specially designated rooms equipped with comfortable beds for sleeping during the study period. To support circadian rhythm synchronization, participants were accommodated in a controlled clinical setting with standardized sleep conditions and exposure to light–dark cycles.

Participation was entirely voluntary, and all subjects were thoroughly informed about the study’s objectives and their right to withdraw at any stage of the research process. Participation was entirely voluntary, based on written informed consent. All subjects were fully informed about the study’s objectives and procedures, and it was clearly emphasized that they could withdraw from the study at any time without providing a reason and without any consequences. The study was conducted in accordance with the principles of the Declaration of Helsinki and was approved by the Bioethics Committee at Pomeranian Medical University in Szczecin (reference number: KB-0012/99/14, approved on 14 December 2022).

### 4.2. Study Material

Venous blood samples were collected from the basilic vein over a single 24 h period, at four time points: 02:00, 08:00, 14:00, and 20:00. The intervals between collections were approximately six hours. All samples were obtained by qualified medical personnel at the University Clinical Hospital No. 2 in Szczecin. Blood was drawn into two types of tubes:

- K_2_EDTA tubes for hematological analysis and plasma preparation.
- Clot activator tubes for serum biochemical and hormonal assays.

Peripheral blood counts were determined immediately after collection using an ABX Micros 60 analyzer (Horiba Medical, France), measuring red blood cell count (RBC), white blood cell count (WBC), hematocrit (HCT), hemoglobin (HGB), and platelet count (PLT).

Serum was separated by centrifugation (2600 rpm, 10 min, 20 °C) within 30 min of collection. All samples were aliquoted into sterile polypropylene tubes and stored at −80 °C until analysis.

Biochemical parameters—including glucose, total protein, albumin, creatinine, uric acid, total cholesterol, HDL, LDL, triacylglycerols (TAG), and minerals (total calcium, phosphorus, magnesium)—were determined using Biomaxima reagent kits (Biomaxima, Lublin, Poland) according to the manufacturer’s instructions. ELISA determined melatonin concentrations within one month of collection, while XOR isoform activities were measured spectrophotometrically within three months of sample storage to prevent enzymatic degradation.

### 4.3. Melatonin Assay

Serum samples collected at four circadian time points (02:00, 08:00, 14:00, and 20:00) were analyzed to verify the rhythmic secretion pattern of melatonin. Melatonin concentrations were determined using a commercial Human Melatonin ELISA kit (Cloud-Clone Corp., Katy, TX, USA) according to the manufacturer’s protocol. Before analysis, serum samples were thawed on ice, gently mixed, and centrifuged (6000 rpm, 5 min, 4 °C) to remove residual particulates. All reagents were equilibrated to room temperature before use. The absorbance was read at 450 nm using an EnVision microplate reader (PerkinElmer, Springfield, IL, USA), and melatonin concentrations were calculated from a standard curve generated from the kit’s provided calibration standards. All measurements were performed in duplicate, and intra-assay variation did not exceed 10%.

### 4.4. Determination of Xanthine Oxidoreductase Activity and Its Isoforms

The total activity of xanthine oxidoreductase (XOR) and its isoforms—xanthine dehydrogenase (XDH), the intermediate dehydrogenase–oxidase form (XDO), and xanthine oxidase (XO)—was determined spectrophotometrically using a PerkinElmer Lambda 40P UV/VIS spectrophotometer (PerkinElmer, Springfield, IL, USA). All measurements were carried out at 30 °C for 5 min. The reaction mixture contained Tris/HCl buffer (pH 7.4), xanthine as a substrate, and plasma as the enzyme source. Changes in absorbance were recorded continuously at the appropriate wavelength.

The correctness of blank selection for XDH, XDO, and XO assays was verified experimentally before the primary analyses. Comparative measurements were performed using three alternative background variants:

(1) buffer + NAD^+^ + xanthine (without plasma),
(2) buffer + NAD^+^ + plasma (without xanthine), and
(3) buffer + xanthine + plasma (without NAD^+^).

The background configuration that produced the most stable baseline and the most accurate correction was selected for each isoform assay. For XDH, the variant excluding xanthine was optimal, for XDO—the exclusion of NAD^+^, and for XO—the exclusion of plasma (Supplementary Materials Figure S1).

#### 4.4.1. Dehydrogenase (XDH) Activity Assay

XDH activity was determined in the presence of NAD^+^ as an electron acceptor.

The reaction mixture contained Tris/HCl buffer (200 µL), NAD^+^ (250 µL), xanthine (500 µL), and plasma (50 µL).

The blank consisted of the same components, but without xanthine, to correct for background NADH formation from non-enzymatic plasma reactions.

The reduction In NAD^+^ to NADH was monitored at 340 nm (ε = 6.22 × 10^3^ L·mol^−1^·cm^−1^).

#### 4.4.2. Dehydrogenase–Oxidase (XDO) Activity Assay

XDO activity reflects the intermediate enzyme form, capable of transferring electrons to both NAD^+^ and molecular oxygen.

The reaction mixture composition was identical to that used for XDH, but absorbance changes were recorded at 302 nm, corresponding to uric acid formation.

The blank sample excluded NAD^+^ instead of plasma, as this configuration better reflects the factual background when monitoring uric acid production at 302 nm.

Oxygen removal by nitrogen purging was tested in pilot measurements, but it did not significantly affect the results.

#### 4.4.3. Oxidase (XO) Activity Assay

XO activity was determined in the absence of NAD^+^, with molecular oxygen serving as the electron acceptor.

The test mixture contained Tris/HCl buffer (450 µL), xanthine (500 µL), and plasma (50 µL).

The blank contained only buffer and xanthine, as recommended for oxidase assays to account for non-enzymatic uric acid formation.

Absorbance changes at 302 nm were recorded over 5 min (ε = 2.30 × 10^3^ L·mol^−1^·cm^−1^).

### 4.5. Statistical Analysis

All data were analyzed using Statistica 13.3 (StatSoft, Kraków, Poland) and Python (StatsModels v0.14) and visualized with Matplotlib v3.8.

Descriptive statistics were expressed as mean ± standard deviation (SD). Normality of distribution was verified with the Shapiro–Wilk test, while homogeneity of variance was tested with Levene’s test. Depending on distribution, comparisons between two groups were performed using either Student’s *t*-test for independent samples or the Mann–Whitney U test. For comparisons across more than two groups, one-way ANOVA or the Kruskal–Wallis test was applied. In the case of multiple pairwise comparisons, the Bonferroni correction was used to adjust the significance threshold.

To assess the effect of time of day (08:00, 14:00, 20:00, 02:00) and sex on xanthine oxidoreductase (XOR) activity and its isoforms, a two-way repeated-measures ANOVA was conducted. When assumptions of sphericity were violated, corrections (Greenhouse–Geisser or Huynh–Feldt) were applied. Post hoc pairwise comparisons were performed with Bonferroni-adjusted tests to control for type I error. Nonparametric alternatives were considered in case of persistent deviations from normality.

Associations between melatonin concentration and XOR isoform activity were examined with Spearman’s rank correlation coefficient (ρ). A significance threshold of *p* < 0.05 (two-tailed) was adopted for all analyses after correction for multiple testing where applicable. Effect sizes were calculated and interpreted according to Cohen’s guidelines (small, medium, large).

Power analyses were performed using G*Power 3.1.9.2 to evaluate the adequacy of the sample size and the risk of type II error. The level of significance was set at α = 0.05 (two-tailed).

For the correlation analyses, post hoc power calculations showed that the observed significant associations were adequately powered:

- In men at 02:00, XO activity was inversely correlated with melatonin concentration (ρ = –0.52, *p* = 0.006, N = 33), with post hoc power ≈ 0.88.
- In women at 14:00, XDO activity correlated significantly with melatonin (ρ = –0.48, *p* = 0.01, N = 33), yielding post hoc power ≈ 0.82.

A sensitivity analysis (two-tailed correlation, α = 0.05, N = 33) indicated that the minimal detectable effect size at 80% power was |ρ| ≈ 0.47. This implies that weaker associations may not have been identified in sex-stratified analyses.

For the repeated-measures design (2 groups × 4 time points, N = 66), sensitivity analysis revealed sufficient power (1 − β ≥ 0.80) to detect medium-sized effects (Cohen’s f ≈ 0.25). However, the study was underpowered to detect minor effects (f ≈ 0.10) reliably. Power depended on within-subject correlations and the degree of sphericity; thus, the presented estimates should be considered conservative.

Overall, the statistical approach ensured reliable evaluation of circadian changes in XOR isoform activity and their associations with melatonin. The inclusion of a Bonferroni correction reduced the risk of false-positive results from multiple comparisons. Power analyses confirmed that the study design provided adequate sensitivity to detect medium-to-large effects, while minor effects might have remained undetected.

To assess circadian rhythmicity of plasma XOR isoforms (XDH, XDO, XO) and melatonin, we fitted a 24 h cosinor model including sex interaction:

y = β0 + β1cos 2πt/24) + β2sin (2πt/24) + β3Sex + β4(Sex × cos(2πt/24)) + β5(Sex × sin (2πt/24)) + ε. y = \beta_0 + \beta_1\cos(2\pi t/24) + \beta_2\sin (2\pi t/24) + \beta_3{\rm Sex} + \beta_4({\rm Sex}\times\cos(2\pi t/24)) + \beta_5({\rm Sex}\times\sin(2\pi t/24)) + \varepsilon. y = β0 + β1cos (2πt/24) + β2sin (2πt/24) + β3Sex + β4(Sex × cos (2πt/24)) + β5(Sex × sin (2πt/24)) + ε.

Sex was coded as 0 = women and 1 = men. Rhythmicity was evaluated by joint Wald tests (β1 = β2 = 0\beta_1 = \beta_2 = 0 β1 = β2 = 0 for women, β1 + β4 = β2 + β5 = 0\beta_1 + \beta_4 = \beta_2 + \beta_5 = 0 β1 + β4 = β2 + β5 = 0 for men).

Amplitude (A) and acrophase (φ, 24 h) were derived from the cosine–sine coefficients.

Confidence intervals for A, φ, and sex-phase differences (Δφ) were obtained using a cluster bootstrap (500 resamples of participants).

## 5. Conclusions

This study demonstrated sex-dependent differences in the activity of xanthine oxidoreductase (XOR) isoforms in healthy adults. Men showed higher total XOR activity and a greater contribution of pro-oxidant forms (XO and XDO). In contrast, women exhibited relatively higher activity of the antioxidant XDH form, indicating sex-specific mechanisms of redox regulation.

Although XOR isoforms did not display statistically significant 24 h rhythmicity, the lowest activity observed in men at night coincided with the melatonin peak, suggesting a potential temporal association between melatonin secretion and enzymatic redox balance. The inverse correlation between XO activity and melatonin levels at 2:00 (*p* = 0.006) further supports a possible link between hormonal and oxidative status. However, the directionality of this relationship remains unclear.

In conclusion, the present findings highlight the modulatory influence of sex on xanthine oxidoreductase activity and suggest that temporal variations in purine metabolism enzymes are subtle and likely reflect broader physiological regulatory mechanisms rather than direct effects of a single factor.

Future studies in clinical populations should investigate whether disruptions in these regulatory patterns contribute to the pathogenesis of cardiovascular, metabolic, or renal disorders and whether accounting for the timing of sample collection could enhance the diagnostic and therapeutic value of oxidative stress markers within a chronobiological context.

## Figures and Tables

**Table 1:** Morphological and biochemical parameters in the study group stratified by sex. Data are presented as mean ± SD.

Parameter	Men (*n* = 33)	Women (*n* = 33)	*p*
RBC [10^12^/L]	5.3 ± 0.5	4.5 ± 0.4	**<0.0001**
HGB [mM/L]	9.2 ± 0.6	7.9 ± 0.6	**<0.0001**
HCT [%]	46 ± 3	40 ± 3	**<0.0001**
WBC [10^9^/L]	5.8 ± 1.1	6.0 ± 1.4	0.9929
PLT [10^3^/µL]	253 ± 40	252 ± 41	0.9715
Glucose [mg/dL]	92 ± 7	90 ± 8	0.1311
Phosphorus [mM/L]	1.5 ± 0.1	1.5 ± 0.2	0.9359
Magnesium [mM/L]	0.8 ± 0.0	0.8 ± 0.0	0.6487
Calcium [mM/L]	2.4 ± 0.1	2.4 ± 0.2	0.7141
Ch–C [mg/dL]	187 ± 22	179 ± 23	0.1555
Triglycerides [mg/dL]	111 ± 35	92 ± 19	0.0207
LDL [mg/dL]	92 ± 25	89 ± 23	0.7141
HDL [mg/dL]	72 ± 14	70 ± 11	0.3669
Total protein [g/dL]	6.6 ± 0.5	6.5 ± 0.4	0.2491
Albumin [g/dL]	4.0 ± 0.3	3.9 ± 0.3	0.0525
Creatynine [mg/dL]	1.1 ± 0.2	1.0 ± 0.2	**0.0012**
Uric acid [mg/dL]	5.3 ± 1.0	4.5 ± 0.7	**0.0005**

Independent samples *t*-test (parametric, normally distributed data, verified by the Shapiro–Wilk test). Mann–Whitney U test (nonparametric). Statistical comparisons between men (*n* = 33) and women (*n* = 33) were performed using the independent-samples *t*-test or the Mann–Whitney U test, depending on the data distribution. Statistical significance was set at *p* < 0.05.

**Table 2:** Cosinor analysis of 24 h rhythms of xanthine oxidoreductase isoforms and melatonin in women and men.

Outcome	Zero amp. p (Women)	Zero amp. p (Men)	MESOR (Women)	MESOR (Men)	Amplitude (Women)	Acrophase [h] (Women)	Amplitude (Men)	Acrophase [h] (Men)	Δ Phase (Men−Women) [h]
XDH	1.32 × 10^−1^	3.71 × 10^−1^	11.62	15.24	2.21	5.62	1.47	4.19	−1.43
XDO	6.88 × 10^−1^	1.17 × 10^−1^	9.64	10.18	0.30	18.53	1.26	0.09	−18.44
XO	4.84 × 10^−1^	2.00 × 10^−1^	11.14	10.75	0.99	19.18	1.34	1.62	−17.56
Melatonin	<1 × 10^−5^	<1 × 10^−5^	33.95	35.42	45.08	3.87	43.65	3.84	−0.03

Amplitude (A). acrophase (φ. in hours), and *p*-values from the zero-amplitude test are shown for each sex and the combined cohort. Δ phase indicates the difference in acrophase between men and women (positive values denote a phase delay in men). MESOR—midline estimating statistic of rhythm; Significant rhythmicity was observed only for melatonin (*p* < 0.001).

**Table 3:** Cosinor analysis of 24 h rhythms of plasma xanthine oxidoreductase (XOR) isoforms in the total study group.

Enzymes	MESOR	Amplitude (Global)	Acrophase (Rad)	R^2^	*p*-Value
XDH	13.43	1.85	1.29	0.012	0.094
XDO	9.90	0.62	0.28	0.005	0.265
XO	10.94	0.78	1.11	0.004	0.375
Melatonin	34.66	44.38	3.85	0.66	<1 × 10^−6^

XDH—xanthine dehydrogenase; XDO—dehydrogenase–oxidase form; XO—xanthine oxidase. MESOR—midline estimating statistic of rhythm; R^2^—goodness of fit; *p*-value—zero-amplitude test (significance of rhythm).

## Data Availability

The datasets presented in this study are available on reasonable request from the corresponding author due to institutional and privacy restrictions. Summary data necessary to reproduce the main findings are included within the article and its Supplementary Material.

## Supplementary Materials

The following supporting information can be downloaded at https://www.mdpi.com/article/10.3390/ijms262311272/s1.
