# Supplementary material for: Redox on the Clock: Sex-Dependent Dynamics of Xanthine Oxidoreductase Isoforms and Melatonin
Source: Int J Mol Sci. 2025 Nov 21;26(23):11272. doi: 10.3390/ijms262311272 (PMC12692483; doi:10.3390/ijms262311272)
Supplement: Supplementary file 1 [file ijms-26-11272-s001.zip › ijms-3908059-supplementary.pdf]

Supplementary Table S1. Statistical parameters for melatonin concentration in women according to the time of material collection [pg/ml].

DESCRIPTIVE STATISTICS OF MELATONIN CONCENTRATIONS IN WOMEN								
Blood Collection Time	Mean	Standard deviation	Median	Minimum	Maximum	Upper quartile	Lower quartile	Interquartile range
**02:00**	99.5	± 11.2	98.4	76.2	135.8	105.3	94.6	**9.4**
**08:00**	15.9	± 3.8	15.3	8.3	22.8	18.2	13.9	**4.3**
**14:00**	8.7	± 2.5	9.1	3.6	13.4	10.3	6.9	**3.4**
**20:00**	**12.9**	**± 4.7**	**12.2**	**4.7**	**27.1**	**15.1**	**9.4**	**5.7**

Supplementary Table S2. Statistical parameters for melatonin concentration in men according to the time of material collection [pg/ml].

DESCRIPTIVE STATISTICS OF MELATONIN CONCENTRATIONS IN MEN								
Blood Collection Time	Mean	Standard deviation	Median	Minimum	Maximum	Upper quartile	Lower quartile	Interquartile range
**02:00**	97.1	± 11.7	98.1	72.1	118.9	104.6	89.4	**15.2**
**08:00**	15.5	± 4.4	14.5	8.2	23.8	18.8	12.0	**6.8**
**14:00**	8.0	± 2.6	8.1	3.9	15.0	10.1	5.8	**4.3**
**20:00**	**12.1**	**± 3.0**	**12.2**	**5.7**	**19.0**	**13.8**	**10.3**	**3.5**

Supplementary Table S3. Descriptive statistics of dehydrogenase activity (XDH) in men according to blood collection time (mU/mL).

Time of collection	Mean	Median	Minimum	Maximum	Lower quartile	Upper quartile	Interquartile range	SD
**02:00**	9.91	7.47	1.55	23.70	5.55	14.35	8.80	6.35
**08:00**	5.36	5.51	3.18	9.13	3.55	6.55	3.00	1.76
**14:00**	7.77	7.21	2.48	14.79	4.81	10.87	6.06	3.49
**20:00**	7.25	6.62	2.92	13.61	4.22	9.47	5.25	3.39

Values are expressed in mU/mL. IQR – interquartile range; SD – standard deviation.

Supplementary Table S4. Descriptive statistics of dehydrogenase activity (XDH) in women according to blood collection time (mU/mL).

Time of collection	Mean	Median	Minimum	Maximum	Lower quartile	Upper quartile	Interquartile range	SD
**02:00**	8.98	7.40	0.85	20.74	4.79	12.87	8.08	5.45
**08:00**	9.00	7.16	1.66	21.23	5.21	12.46	7.25	5.36
**14:00**	8.73	6.18	1.77	21.71	4.40	13.05	8.65	5.65
**20:00**	7.46	6.75	0.63	16.42	4.36	9.50	5.14	4.39

Values are expressed in mU/mL. IQR – interquartile range; SD – standard deviation.

Supplementary Table S5. Descriptive statistics of xanthine dehydrogenase/oxidase (XDO) activity in men according to blood collection time

Time of collection	Mean	Median	Minimum	Maximum	Lower quartile	Upper quartile	Interquartile range	SD
**Time of collection**	Mean	Median	Minimum	Maximum	Lower quartile	Upper quartile	Interquartile range	SD
**02:00**	8.88	8.80	0.71	19.22	2.79	13.31	10.51	6.37
**08:00**	19.22	16.31	9.37	32.24	14.41	25.21	10.81	7.53
**14:00**	17.93	19.71	0.74	44.61	4.13	28.88	24.74	14.21
**20:00**	2.38	1.87	0.58	5.27	0.90	3.59	2.68	1.11

Values are expressed in mU/mL. IQR – interquartile range; SD – standard deviation.

Supplementary Table S6. Descriptive statistics of xanthine dehydrogenase/oxidase (XDO) activity in women according to blood collection time

Time of collection	Mean	Median	Minimum	Maximum	Lower quartile	Upper quartile	Interquartile range (IQR)	Standard deviation (SD)
**02:00**	12.58	11.79	4.20	25.84	5.33	18.74	13.41	7.10
**08:00**	7.54	7.04	2.84	14.86	5.56	9.69	4.13	3.33
**14:00**	6.59	5.38	0.94	15.51	4.17	9.98	5.81	4.08
**20:00**	14.56	13.41	3.13	33.92	7.24	19.71	12.47	10.17

Values are expressed in mU/mL. IQR – interquartile range; SD – standard deviation.

Supplementary Table S7. Descriptive statistics of xanthine oxidase (XO) activity in men according to blood collection time.

Time of collection	Mean	Median	Minimum	Maximum	Lower quartile	Upper quartile	Interquartile range (IQR)	Standard deviation (SD)
**02:00**	7.32	4.33	0.13	25.39	1.68	11.86	10.18	7.51
**08:00**	25.16	16.09	2.91	70.91	10.66	34.98	24.32	20.10
**14:00**	44.02	25.52	0.71	136.64	2.58	86.25	83.67	45.04
**20:00**	24.92	8.85	0.31	91.61	3.07	48.78	45.71	27.87

Values are expressed in mU/mL. IQR – interquartile range; SD – standard deviation.

Supplementary Table S8. Descriptive statistics of xanthine oxidase (XO) activity in women according to blood collection time

Time of collection	Mean	Median	Minimum	Maximum	Lower quartile	Upper quartile	Interquartile range (IQR)	Standard deviation (SD)
**02:00**	14.46	12.92	2.58	31.66	8.03	21.16	13.13	8.04
**08:00**	8.09	6.46	1.97	14.86	4.62	12.28	7.66	4.42
**14:00**	6.19	6.14	1.13	10.11	4.52	9.04	4.52	2.76
**20:00**	7.71	8.08	1.94	15.18	5.81	9.69	3.88	3.66

Values are expressed in mU/mL. IQR – interquartile range; SD – standard deviation.

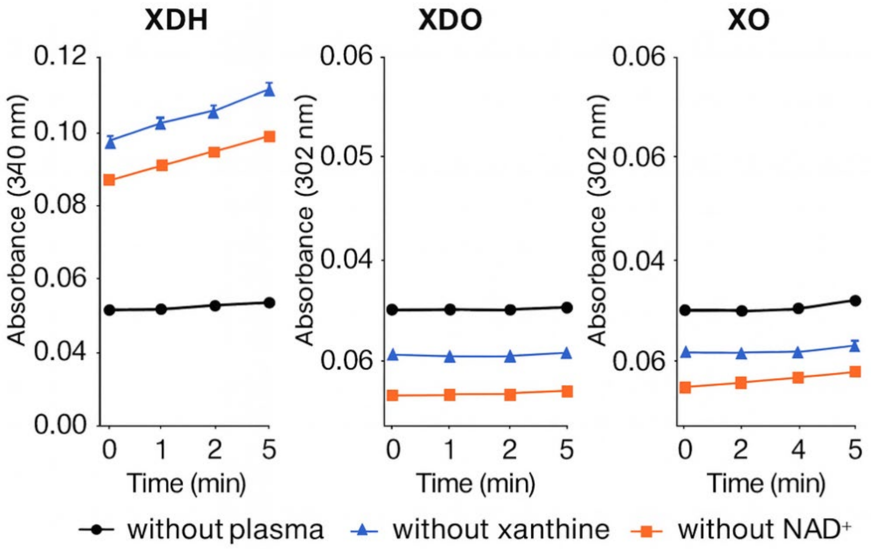

Figure S1. Comparison of blank variants in XOR isoform assays. Time-dependent changes in absorbance (340 nm for XDH, 302 nm for XDO and XO) recorded over 5 min. Each line represents a different blank configuration.
